# Supplementary material for: A scoping review of interventions on middle school students’ attitudes towards science
Source: PLoS One. 2025 Jan 14;20(1):e0315757. doi: 10.1371/journal.pone.0315757 (PMC11731865; doi:10.1371/journal.pone.0315757)
Supplement: S1 Protocol — (DOCX) [file pone.0315757.s001.docx]

**S1 File. Protocol of the systematic review**

**A scoping review of interventions on middle school students' attitudes towards science**

**Objectives of the review:**

The aim of this review is to examine the existing scientific evidence in order to provide a comprehensive description of the characteristics of educational training programmes aimed at fostering attitudes towards science in secondary school students.

To this end, the guiding question of the review is articulated below.

- What is known in the existing literature about educational training programmes aimed at promoting attitudes towards science in secondary school students?

In order to provide a more complete overview of the literature to be addressed, the following specific questions will also be answered.

- How has the construct of attitudes towards science been addressed in the literature in terms of focus, references of origin and dimensionality of the scale?
- What are the characteristics of the reported educational interventions in terms of teaching approach, duration, implementer, intervention context, impact on attitudes and monitoring of programme development?
- What were the main problems and recommendations identified by the researchers within the localised educational interventions?

In addition to the research questions presented previously, the research team aimed to examine the current status of localised training programmes in terms of sociodemographic and methodological information (country of origin, year of publication, research design and methodology, number of participants and factors influencing students' attitudes towards science). This information will help us to provide an overview of the information analysed in the current study.

**Background:**

Despite the paramount importance of science and technology for societal progress, there is a discernible trend among students in basic education that their interest in this field of knowledge wanes as they progress through their academic careers. As a result, the cultivation of positive attitudes towards science becomes increasingly important. The implementation of multidisciplinary programmes has the potential to facilitate, to some extent, the achievement of positive attitudes towards science among secondary school students.

**Inclusion criteria**

The criteria used to conduct the scoping review are listed below. Where indicated, their inclusion is justified by the objective of the study.

- Secondary school students aged 12 to 16.
- Attitudes towards science (covers attitudes towards disciplines such as biology, physics or chemistry.
- Studies that involve the implementation of a training programme or educational action towards students.
- Studying in English or Spanish.
- Within the last 10 years.

The study focuses on secondary school students, as defined by the Mexican Ministry of Public Education (Secretary of Foreign Affairs [SRE], 2021), which includes individuals between the ages of 12 and 16. Therefore, the study population includes students in this age group enrolled in secondary education, which includes different modalities such as general, technical, telesecundaria, community (General Law of Education [LGE], 2019), or analogous modalities, according to the criteria established in the different countries of origin of the found documents.

With regard to the construct of attitudes towards science, it goes beyond the mere identification of primary documents that explicitly use this expression. It includes attitudes towards scientific disciplines that are an integral part of science education, in particular chemistry, physics or biology. It is important to note that this inclusion criterion may potentially exclude relevant documents due to its limitation to specific disciplines, although these are limited to areas of interest outlined in the secondary school curriculum in Mexico (SEP, 2022).

It is considered imperative to evaluate the outcomes or impacts of training programmes or initiatives aimed at fostering attitudes towards science. Training programs or initiatives refer to the structured educational processes aimed at cultivating new competencies (UNESCO, 2019), particularly in relation to the aforementioned construct. The scope extends not only to classroom-based initiatives, but also to those that take place outside the conventional classroom or school setting, provided that they adhere to the defining characteristics of educational training. This section seeks to identify guidelines for the delineation of potential interventions, as well as potential problems, barriers or constraints that could hinder the success of educational efforts aimed at fostering attitudes towards science.

Furthermore, for reasons of practicality, database availability and efficiency of document interpretation, the search is limited to studies or documents written in English and Spanish. The search is also limited to studies conducted within the last ten years. This timeframe was chosen in order to provide a comprehensive, contemporary overview of such studies, in line with the rationale articulated in this section, and to ensure a more exhaustive exploration of the relevant literature.

**Context**

The study is not limited to a specific geographical region, provided that the documents are available in the languages indicated. In addition, it emphasises the importance of prioritising the study of socio-economic factors within the defined population characteristics.

**Sources**

A comprehensive review of various research databases will be conducted, including Eric, Google Scholar, Dialnet, Scielo, EbscoHost, Scopus and Web of Science. Search queries will be formulated to access different types of research articles, including review articles, scientific articles, case studies and theoretical papers. In addition, efforts will be made to access dissertations or handbooks related to the topic under study. It is important to note that the search will not be limited to peer-reviewed literature. However, it is important to emphasise that opinion pieces, conference proceedings or websites will be excluded from consideration.

**Search strategy**

As outlined by Peters et al. (2020), the search for primary documents will involve three main stages, which are explained as follows:

1. **Initial database search**: The search for documents begins within databases relevant to the topic, using pre-defined search queries. In this phase, at least two online databases will be searched in order to refine the initial search queries by examining the titles, keywords and abstracts of relevant studies. This process will be carried out in a Spanish database (SciELO) and an English database (ERIC) in order to identify key concepts that are relevant in different contexts.
2. **Subsequent search**: Following the refinement of the initial search queries, a subsequent search for documents will be carried out across databases. In addition to those mentioned above, online databases such as Google Scholar, Dialnet, EbscoHost, Web of Science and Scopus will be included. The aim is to identify documents that meet the established inclusion criteria. It is plausible that further searches or updates may be necessary based on the emergence of inclusion/exclusion criteria.
3. **Evaluation of findings**: The list of documents obtained from previous searches is then examined and a manual selection is made to form the basis of the analysis. This involves evaluating the results from each database, focusing only on full documents rather than web page links.
4. **Additional literature review**: As a complementary measure, relevant literature will be searched within the references provided in the documents retrieved from the databases. The aim of this exercise is to add valuable information to the study.

Following this process, the comprehensive search strategy is meticulously documented in each of the online databases consulted. This documentation will highlight the date of the search, the version of the search, the query used, the inclusion/exclusion criteria implemented during refinements, the publication period considered and the results obtained in each iteration.

**Data extraction**

A summary data table is used to extract relevant information. The aim of this table is to systematically organise key findings in order to effectively address all research enquiries. The table is included as S2 File.
