# Supplementary material for: A scoping review of interventions on middle school students’ attitudes towards science
Source: PLoS One. 2025 Jan 14;20(1):e0315757. doi: 10.1371/journal.pone.0315757 (PMC11731865; doi:10.1371/journal.pone.0315757)
Supplement: S1 Appendix — (DOCX) [file pone.0315757.s003.docx]

**S3 File. Table of data concentration**

**Category A. General information about the study**

1.       Title of the study

2.       Author(s)

3.       Country in which the study was developed

4. Region in which the study was developed

a.       Europe

b.      North America

c.       Central & South America

d.      Australasia

e.      Asia

f.    Africa

g.       Multiple regions

h.      Not mentioned

5.     Year of publication

6.     Study design

a.       Quantitative

b. Qualitative

c. Mixed

7. Method

8.       Aim of the study

**Category B. Information on the construct to be analysed**

9.    Does the study define the construct of interest?

a.       Yes

b.      No

10.   Interest construct approach

a.       Attitudes to science in general

b.      Attitudes towards a science course

c.       Attitudes towards a scientific discipline

                                                               i.      Science Physical

                                                             ii.      Chemistry

                                                            iii.      Biology

d.      Attitudes to a particular issue

e.      Other

11.   Theory on which the construct is based

12.   Formation of the construct

a.       Unidimensional

b.      Multidimensional

13.   Which dimensions are addressed?

14.   Are the results compared per construct dimension?

a.       Yes

b.      No

15.   Is the attitude construct the main variable of the study? (Based on what is stated in sections such as: Introduction, Theoretical References or Problem Statement)

a.       Yes

b.      No

16.   Type of instrument used to assess students' attitudes

a Questionnaire/scale

b.    Interview

c.    Observation tool

d.    Focus group

e.      Other

17.   Was there a follow-up evaluation to assess the sustainability of the attitudes?

a.      Yes

b.      No

**Category C. Information on the participants**

18.   Number of participants

19.   Age

20. Year in school

21.   Characteristics of interest that are mentioned in the studies (intermediate variables)

**Category D. Information on the intervention developed**

22.   Teaching method used (as indicated by the authors)

23.   Type or typology of intervention

a.       Context-based teaching

b.      Inquiry-based learning

c.       Learning environments enriched with computer technology-based instruction

d.      Collaborative learning

e.      Extracurricular activities

f.     Questioning strategies

g.    Focus strategies

h.    Manipulation Strategies

i.     Enhanced material strategies

j.    Evaluation strategies

k.   Direct instruction

l.        Other

24.   Duration of intervention

25.   Implementer

a.       Teacher

b.      Researcher-research team

c.       Other

26.   Previous training of the person who applied the intervention?

a.       Yes

b.      No

27.   Is there a tool or procedure in place to evaluate the programme design?

a.       Yes

b.      No

28.   Is there a tool to evaluate the implementation of the programme according to the objectives or intervention guidelines?

a.    Yes

b.      No

29.   Location of the intervention

a.       Within the school

b.      Out of school

c.       A mixture of the two

30.   Is intervention on attitudes justified from any point?

a.       Yes

b.      No.

31.   Did the proposed intervention help to develop attitudes towards science?

a.       Yes

b.      No

32.   Other findings

33.   Problems during the training mentioned by the authors

34.   Training recommendations from the authors
