# Supplementary material for: A scoping review of interventions on middle school students’ attitudes towards science
Source: PLoS One. 2025 Jan 14;20(1):e0315757. doi: 10.1371/journal.pone.0315757 (PMC11731865; doi:10.1371/journal.pone.0315757)
Supplement: Attachment — Submitted filename: Reviewers report_27.08.24.docx [file pone.0315757.s004.docx]

**Reviewer’s report**

The manuscript has certainly showcased extensive work on data collection and highlighted issues about educational interventions to promote positive attitudes towards science among middle school students, such as several gaps in the understanding of the constructs used in these interventions as well as the need to further research to focuses on intrinsic factors like racial background and perceptions on self-efficacy to better understand the long-term impact of these educational programs. Nonetheless, some issues in the presentation and discussion of the data should be addressed by the authors before publication so that the results presented in the manuscript can be better understood. Therefore, the following questions should be addressed by the authors:

1. In the Abstract (page 2, lines 6-8), the authors state “This paper presents a scoping review of 37 papers published in English and Spanish on the intervention literature from the last 10 years, both to describe the research outputs and to analyse the evidence on the effectiveness of different scientific databases”. I am not sure what the authors mean by “effectiveness of diferente scientific databases”. Please clarify.

2. Please revise the references format throughout the text. For example, in the introduction section (page 3, lines 11-12) reads: “Guo et al., 20-22; Musengimana et al., 20-21”. The hyphen should be removed between the year-numbers.

3. Still on page 3 (lines 16-18), the authors states: “In the words of Helvaci & Yilmaz (2022), the pedagogical practices developed by teachers have an impact on students' motivation and therefore promote the formation of positive attitudes towards science”. By the choice of wording by the authors, it is understood that the statement is a direct citation from Helvaci & Yilmaz. If so, quotation marks are needed.

4. In Table 1 the authors describe the pedagogical approaches used in science education. However, it is not clear within the table with theorical references were used to define each approach. I suggest revising the table to clarify this issue.

5. On page 6, the caption of Table 1 reads “Note. Original creation”. I suggest changing it to “Table created by the authors”. Same goes for the other tables in the manuscript.

6. On page 8 (lines 4-7), the authors state: “In the words of Savelsbergh et al. (2016), studying the constructs together may lead to confusing interpretations of the outcomes of an intervention, making it relevant to address them independently”. By the choice of wording by the authors, it is understood that the statement is a direct citation from Savelsbergh et al. If so, quotation marks are needed.

7. Still on page 8 (lines 10-13), reads: “Therefore, it may also be appropriate to analyse only those educational interventions that are implemented at the appropriate age for secondary education. For this reason, it may also be appropriate to analyse only those educational interventions that are implemented at the appropriate age for secondary education”. The phrase is duplicated. Please revise.

8. In the Method section (page 9), the authors describe the use of a “initial search equation” to explore concepts that would apply to the proposed scoping review. However, it is not clear throughout the manuscript nor in the supplementary information what these search equations were. Please clarify. The searching terms should be clearly described in the methodology section.

9. Regarding the figures and tables, all the figures and tables in the manuscript are presented without a caption. Figures should be followed by a descriptive caption describing the data shown and drawing attention to relevant findings or observations. Please revise.

10. Regarding Figure 3, I suggest revising the way the data is presented. The number of publications for each country should appear in the figure or be described in the caption (see comment above).

11. On page 18 (lines 6-7), reads: “The analysis shown in Figure 3 does not include documents that aim to conduct a literature review”. I believe that the referred analysis is the one shown in figure 4 and not figure 3 (as stated in the text). Please, revise.

12. Regarding Figure 4, I suggest including the number of each type of research design in the graph along with the percentages. Also, the caption is missing (see comment 9).

13. Figure 5, it is not clear what the category “ATPT” means, the caption is incomplete. It reads: “ATPT= Attitudes toward a particular”. Should it be “particular topic”? If so, what is the difference between this category and the previous one, ATSCD (particular science discipline)? Please clarify. Moreover, I suggest include the numbers related to each category in the graph and in the caption (see comment 9).

14. Figure 6, I suggest including the numbers on top of the bars in the graph to facilitate the reading of the data. Caption is also needed (see comment 9).

15. In the Discussion section, I encourage the authors to dig deeper into their interpretation of the data collected from their review, especially those regarding racial/ethnic identity and the lack of studies focusing on the topic. For example, the authors could explore the implications of those interventions that are under-researched, delving into why that might be the case and how it impacts practice or policies in science education. I strongly believe that exploring those points would benefit the discussion of the manuscript.

16. Also in the Discussion section (page 30, lines 19-22), reads: “Notably, our study reveals that only seven documents acknowledged the use of evaluation tools to assess programme implementation against objectives or intervention protocols, with only four considering tools or methods to assess programme design.” It should be stated which studies are the ones mentioned. Please revise.

17. In the Discussion section (pages 26-30) and later in the Conclusions (page 30-32) the authors highlight several limitations of their review. I suggest the authors to include a Limitation section after the conclusions so that they can explore in more details the implication of those limitations and how future studies could undermine such limitations.

18. In the supplementary 1 (attached to the manuscript), the authors describe their review protocol. In the protocol, one of the specific research questions is: “What is the current status of localised training programmes in terms of socio-demographic variables?’. However, in the description of the research questions in the manuscript (page 8), the above question is not mentioned. The question doesn’t appear to be addressed by the results presented in the manuscript either. Please, clarify.

19. Also in the supplementary 1, in the “Data extraction” section, it reads: “A summary data table is used to extract relevant information. The aim of this table is to systematically organise key findings in order to effectively address all research enquiries. The components to be included in the table are outlined below”. However, no table is presented. Please, provide table.

I would be happy to review a revised version of this manuscript.
